# Emotional Modulation of the Pupil Response in Psychopathy

**DOI:** 10.1037/per0000313

**Published:** 2018-12-03

**Authors:** Daniel T. Burley, Nicola S. Gray, Robert J. Snowden

**Affiliations:** 1 School of Psychology, Cardiff University; 2 Department of Psychology, Swansea University, and Department of Psychology, Abertawe Bro-Morgannwg University Health Board, Swansea, Wales; 3 School of Psychology, Cardiff University

**Keywords:** psychopathy, interpersonal–affective, emotion, pupillometry

## Abstract

Psychopathy is a form of personality disorder associated with a deficit in emotional processing. However, there is debate whether this deficit applies to all emotions or exists only for negative emotions. The pupil dilates rapidly in response to emotional stimuli, allowing a time-sensitive index of emotional processing. Across 3 experiments using (a) visual images of real-world scenes, (b) auditory sound clips, and (c) videos of dynamic facial expressions, we measured emotional modulation of the pupil response to both negative and positive stimuli. Participants were 82 male mentally disordered offenders. Psychopathy was measured using the Psychopathy Checklist—Revised to produce factor scores of interpersonal–affective traits (Factor 1) and lifestyle–antisocial traits (Factor 2). Participants with high Factor 1 scores showed reduced emotional modulation of the pupil response to negative images and angry faces but not to any of the positive stimuli. These effects only occurred shortly after the emotion was presented (<2,000 ms), suggesting delayed processing of negative affective stimuli in Factor 1 psychopathy. Factor 2 scores were not associated with any changes in pupil response. There were no effects of psychopathy on the pupil response to the affective sound clips. The results support a specific psychopathic deficit in the processing of negative stimuli related to the interpersonal–affective dimension of psychopathy. We argue that pupillometry is a powerful and noninvasive tool to investigate emotional processing in clinical populations.

Psychopathy is a form of personality disorder that is associated with pervasive antisocial behavior. Many theories of psychopathy propose an affective deficit, where stimuli that contain affective content fail to induce normative affective states, behavioral responses, or physiological reactions (Hare, 2003). However, the evidence for the nature of this emotional deficit underpinning psychopathy is inconsistent (Brook, Brieman, & Kosson, 2013; Dawel, O’Kearney, McKone, & Palermo, 2012). A general emotional deficit perspective argues that psychopathy is linked to a blunted capacity for experiencing all emotions (Cleckley, 1941). There is some evidence to support such a position. For instance, Verona, Patrick, Curtin, Bradley, and Lang (2004) found that psychopathy was related to a deficit in galvanic skin responses to affective sounds for both negatively valenced and positively valenced sounds. Others, however, suggest that psychopathy is associated with a problem in processing only negative emotions such as sadness and fear. For example, the low-fear hypothesis (Lykken, 1957) proposes that psychopathy is characterized by a reduced capacity for activating fear systems, leading to an insensitivity to fearful cues.

There is evidence to support a negative-specific deficit within psychopathy. For example, Benning, Patrick, and Iacono (2005) reported that individuals high in psychopathy evidenced deficient skin conductance magnitudes only in response to aversive images. Further, Patrick, Bradley, and Lang’s (1993) seminal work examined how the startle response to a loud stimulus is altered when viewing affective images. For individuals low in psychopathy, aversive pictures increased the magnitude of the startle response, whereas positive images decreased the startle reflex. However, for those with high psychopathy scores, the aversive images did not produce the expected increase in the startle response (and may even have caused a decrease). In contrast, the positive images produced a typical decrease in startle reflex. Hence, this experiment points to a selective deficit in processing the aversive affective content of stimuli for individuals high in psychopathy.

However, Brook et al. (2013) concluded in their review that the “overall pattern of findings is not clearly consistent with any of the dominant theoretical perspectives of emotion processing in psychopathy” (p. 979). It seems probable that different experimental paradigms, which use different stimulus types (e.g., image-based, auditory, and facial stimuli) and that have different task demands, have all contributed to this inconsistent pattern of results. Therefore, it seems pertinent to explore a basic autonomic response to affective cues (both negative and positive) across a range of stimulus modalities to further evaluate the nature of emotional processing impairments associated with psychopathy.

## Pupillometry

The pupil is known to be sensitive to the affective content of a stimulus. Although some early studies suggested that negative stimuli cause a constriction of the pupil and positive stimuli cause a dilation (Hess & Polt, 1960), many recent studies show that both negative and positive stimuli produce a significant dilation, and this has been demonstrated for visual images, sound clips, and facial stimuli (Bradley, Miccoli, Escrig, & Lang, 2008; Bradley, Sapigao, & Lang, 2017; Burley, Gray, & Snowden, 2017; Partala & Surakka, 2003; Snowden et al., 2016). Hence, it appears that it is not the valence of a stimulus that causes the dilation but instead the arousal induced by the stimulus. However, the exact nature of which affective stimuli produce pupil dilation is still debated. Some studies have looked at how people’s subjective ratings of a stimulus are related to pupil dilation (Bradley et al., 2017; O’Farrel, 2016). Although there is a relationship between subjective rating and pupil dilation, it is also clear that some stimuli that are subjectively rated as arousing do not give rise to a corresponding degree of pupil dilation (such as pictures of cute animals and babies). Instead, it is those stimuli that would normally demand immediate action if encountered in real life (e.g., threats, sex, and violence) that cause the greatest pupil dilation. This has led to the suggestion that the degree of pupil dilation is related to the extent to which the stimulus engages the fundamental defensive or appetitive motivational systems (Bradley et al., 2017).

At a physiological level, pupil diameter is mediated via the activity of two groups of muscles: the sphincter pupillae and the dilator pupillae. The sphincter pupillae are controlled by the parasympathetic nervous system and activation serves to constrict the pupil. The dilator pupillae are innervated by the sympathetic nervous system and activation of these muscles serves to dilate the pupil. It is this sympathetically mediated dilation that is being detected in experiments using affective stimuli (Bradley et al., 2017). The sympathetic activity arises from activation of the amygdala, and pupil dilation is considered to reflect amygdala activity given that direct stimulation of the amygdala leads to pupil dilation (Koikegami & Yoshida, 1953), and there is evidence for the co-occurrence of both pupil dilation and increased amygdala activity (Siegle, Steinhauer, Thase, Stenger, & Carter, 2002).

It is clear that the amygdala plays a major role in the processing of threat stimuli and the mediation of fear and is important in fear conditioning (Phelps & LeDoux, 2005). Hence, a dysfunction in the amygdala may cause someone to lack fear and to fail to learn from punishment. Not surprisingly, dysfunction of the amygdala has been put forward as a possible neurophysiological explanation of many features of psychopathy (Blair, 2003). However, the amygdala is also sensitive to many other stimuli, including positively valenced stimuli. For example, Hamann, Ely, Hoffman, and Kilts (2002) showed strong activity in the amygdala, via positron emission topography, to a mixture of positive images showing nudes, appealing animals, infants, and foods. They also showed that the amygdala was responsive to unusual or interesting images. Such findings suggest the amygdala is involved in processing the “motivational salience” of stimuli (Cunningham & Brosch, 2012), including both negative and/or positive stimuli. Therefore, the pupil offers a noninvasive index of amygdala activity, which is valuable to investigate the emotional deficit underpinning psychopathy given the proposed link between psychopathy and abnormal amygdala structure and function (Moul, Killcross, & Dadds, 2012).

There are several pragmatic advantages to pupillometry compared with other measures of sympathetic activity, such as skin conductance response (SCR). First, pupil dilation is far more dynamic than the slow response of the palmar sweat glands and may provide a more time-sensitive psychophysiological tool to explore autonomic reactivity to emotional stimuli. In addition, the pupil response is a more reliable measure of emotional arousal. Bradley et al. (2017) reported that 92% of their sample of healthy volunteers had significantly larger pupils to emotional images compared with neutral images, whereas only 67% of the participants evidenced greater SCR to the same images. Importantly, the pupil’s reaction to the affective component of stimuli is automatic and is not influenced by habituation or attention (Snowden et al., 2016).

There has been one previous study examining the effects of psychopathy on the emotional modulation of the pupil response (EMPR; Burley et al., 2017), but this did not demonstrate any significant effects of psychopathy on pupil responsivity to negative and positive stimuli across a range of stimulus types. However, this study used an undergraduate sample and measured psychopathy using a self-report questionnaire. We aimed to build upon this study to investigate the emotional deficits in psychopathy by measuring psychopathy using the Psychopathy Checklist—Revised (PCL-R; Hare, 2003) in a forensic population where a proportion of individuals were expected to show elevated levels of psychopathic traits.

## Factors and Facets of Psychopathy

Psychopathy has been traditionally considered as a unitary construct with a single underlying cause (Neumann, Hare, & Newman, 2007), but research suggests that this approach may mask the contribution of underlying dimensions, and therefore, psychopathy has been explored as a multifaceted disorder. The PCL-R, the instrument most often used within offender/criminal samples and in clinical settings, is thought to be underpinned by two factors: Factor 1 (interpersonal–affective) capturing features such as grandiosity, lack of guilt, and callousness, and Factor 2 (lifestyle–antisocial) capturing impulsive and disinhibited antisocial behavior. Factor 1 has been found to be associated with deficits in processing emotions, including blunted physiological responses to affective stimuli. For example, in the study of Patrick et al. (1993) described earlier, the lack of potentiated startle to the aversive stimuli was only associated with Factor 1 and not Factor 2 (see also Vaidyanathan, Hall, Patrick, & Bernat, 2011; Vanman, Mejia, Dawson, Schell, & Raine, 2003). More recent models have further separated the PCL-R factors into a four-facet model of psychopathy (Hare, 2003), with interpersonal (Facet 1), affective (Facet 2), lifestyle (Facet 3), and antisocial (Facet 4) facets. This highlights the importance of carefully considering both factor and facet models of psychopathy when investigating the nature of the emotional processing deficit underpinning psychopathy.

## Current Study

The current study aimed to define the nature of emotional processing impairments associated with psychopathy by measuring pupil responses to negative, positive, and neutral stimuli across three different modalities (images, sounds, and dynamic facial expressions) within a population of offenders with a mental disorder. Psychopathy was indexed via the clinician-rated PCL-R. We hypothesized that Factor 1 of the PCL-R would be related to reduced pupil dilation to negative (and possibly positive) stimuli, whereas Factor 2 would be unrelated to pupil responses to emotional stimuli.

Theories of affective deficits (Blair, Mitchell, & Blair, 2005) or of attentional deficits (Newman & Lorenz, 2003) in psychopathy both predict a reduction in affective processing that could be overcome if the stimulus was presented for a sufficient time or at a sufficient intensity (Hamilton, Hiatt Racer, & Newman, 2015). Hence, it seems likely that deficits due to psychopathy might be more evident “early” in the response to a stimulus, with the deficit weakening or disappearing at a “late” period, although the precise definition of early and late is not possible, as this is likely to vary with factors such as stimulus intensity and modality. Nevertheless, we hypothesized that deficits are more likely in an early period than in a later period. Indeed, several studies have found that psychopathic individuals demonstrate attenuated physiological responses to emotional stimuli early in the sequence of responses (up to around 2,000 ms), but normal later responses (Justus & Finn, 2007; Levenston, Patrick, Bradley, & Lang, 2000; Sutton, Vitale, & Newman, 2002). Hence, all analyses examined the effects of emotional content on pupil response over the time course of each stimulus with the hypothesis that the effects of psychopathy would be stronger earlier in the pupil response.

## Method

### Participants

Eighty-two men (*M*_age_ = 38.6, *SD* = 12.8) were recruited from low and medium secure psychiatric hospitals in the United Kingdom. The majority had a diagnosis of a personality disorder (*n* = 68, 83%), with other common diagnoses being schizophrenia and schizotypal and delusional disorders (*n* = 40, 49%) and mood disorders (*n* = 8, 10%). Table 1 presents their demographic information.[Table-anchor tbl1]

Sample size was based on an a priori power calculation for a linear multiple regression including two predictor variables (Factor 1 and Factor 2) with 80% power (α = .05) to detect a medium effect size (*f*^2^ = 0.15). Participants gave written informed consent to participate in the experimental procedures and for the research team to access their hospital medical records. They were paid for their participation. All experimental procedures were given National Health Service (NHS) ethical approval (Research Ethics Committee reference: 14/SC/1198).

Participants were eligible to take part in the study if they had the ability to give informed consent and were not currently psychotic. The responsible clinician^1^ for each patient made these judgments. Only participants with an intelligence quotient (IQ) above 70 took part. Participants were free from any documented history of head injury (defined as a loss of consciousness for more than 1 hr), spoke English as their first language, and had normal/corrected-to-normal vision. Random drugs tests carried out by the hospitals during the research period to test for illicit substance misuse were negative for all cases.

It was recorded whether participants were taking antipsychotic, antianxiety, and/or antidepressant medication, with 32.9% of the sample being medication-free. Across the sample, 61.0% of the participants were taking antipsychotic medication (atypical only = 46.3%, typical only = 11.0%, and both atypical and typical = 3.7%), 26.8% were taking benzodiazepines, and 20.7% were taking antidepressant medication (see the online supplemental materials for further details).

### Materials and Design

Affective images, affective sounds, and dynamic facial expressions were presented across three independent tasks in this set order. Stimulus presentation order within each task was randomized. Participants were read these instructions before each task, “You are now going to be presented with some images/sound clips/video clips. Your task is to pay attention to them, and keep your eyes on the screen while keeping your head as still as you can.”

#### Affective images

Thirty images from the International Affective Picture System (Lang, Bradley, & Cuthbert, 2008) were selected. Ten were negative (mean valence/arousal based on International Affective Picture System ratings = 2.91, 6.20), 10 were positive (7.60, 5.89), and 10 were neutral (5.17, 3.10) images. All images were presented in grayscale and matched for luminance and contrast. Each image was presented for 2,000 ms and was preceded by an equiluminant gray screen displaying a fixation cross (for 2,000 ms) and was followed by a similar plain gray screen for 5,000 ms.

#### Affective sounds

Thirty sound clips were selected from the International Affective Digital Sounds (IADS; Bradley & Lang, 2007), of which 10 were negative (mean valence/arousal = 2.87, 7.09), 10 were positive (7.17, 6.76), and 10 were neutral (5.06, 5.05). The sound clips did not differ (*p*s > .25) across valence categories for average (*M* = −22.61, *SD* = 2.52) or maximum root-mean-square decibel level (*M* = −11.61, *SD* = 2.65). Sound clips were played to participants at a comfortable set volume through headphones. The sound clips were presented for 6,000 ms and presentation order was randomized. A gray fixation slide was displayed throughout and was presented for another 10,000 ms post offset of auditory stimulus. This recovery time was based on preliminary pilot work and allowed for the pupil to return to baseline levels before the next trial began.

#### Dynamic facial emotions

Forty-eight video clips were selected from the Amsterdam Dynamic Facial Expression Set (van der Schalk, Hawk, Fischer, & Doosje, 2011) comprising four male and four female actors simulating facial expressions of fear, happiness, neutral, and anger. We chose to use dynamic facial stimuli because previous research has shown that these are more effective than static emotional expressions of faces for portraying emotions (Alves, 2013). The videos were presented for 4,000 ms and depicted an actor displaying a neutral face before changing into the target expression commencing 500 ms post video clip onset and taking 500–1,000 ms to reach full expression (van der Schalk et al., 2011). Screenshots were taken from the end of each video clip and luminance, contrast, and color showed no differences between valences (*p*s > .35). All actors were presented facing forward and with direct gaze. Each video clip was preceded for 2,000 ms and followed for 5,000 ms by a gray screen luminance-matched to the video clips.

### Psychopathy Checklist—Revised

The PCL-R (Hare, 2003) is a clinician-rated measure of psychopathy. Factor 1 represents interpersonal–affective psychopathy traits (e.g., grandiosity, callousness/lack of empathy, and shallow affect), whereas Factor 2 reflects the lifestyle–antisocial dimension of psychopathy (e.g., impulsivity, parasitic lifestyle, and criminal versatility). PCL-R also provides facet-level scores across the interpersonal (Facet 1), affective (Facet 2), lifestyle (Facet 3), and antisocial (Facet 4) facets.

Across the sample, 31.7% of the participants had been assessed within the past 5 years using both interview and file review by a trained clinical/forensic psychologist. The remaining 68.3% of the participants were assessed on the PCL-R through a collateral file review. Reliable and valid PCL-R ratings can be made on the basis of file information alone that are in line with scores obtained from full PCL-R assessments (Hare, 2003). The rater (D. B.) was trained on the administration of the PCL-R and had completed the Darkstone Post-Workshop Training Program with an interclass correlation of .87. As a check of interrater reliability, the rater conducted a file review on three participants who had been previously assessed using the full PCL-R assessment, achieving an intraclass correlation of .96 for total PCL-R score. Within the current sample, Factor 1 and Factor 2 demonstrated high internal consistency (Factor 1, α = .84; Factor 2, α = .78), although item scores were not available for six participants.

### Wechsler Abbreviated Scale of Intelligence

Intelligence was measured using the two-subtest form of the Wechsler Abbreviated Scale of Intelligence (Wechsler, 1999) that gives an estimate of full-scale IQ. Twenty-six participants had previously completed the Wechsler Adult Intelligence Scale—third edition or the Wechsler Adult Intelligence Scale—fourth edition (Wechsler, 2014), and so, this score was recorded from their patient notes as a more comprehensive assessment of intelligence. IQ scores were similar across the Wechsler Abbreviated Scale of Intelligence (*M* = 90.14, *SD* = 12.88) and the Wechsler Adult Intelligence Scale—third edition/Wechsler Adult Intelligence Scale—fourth edition (*M* = 87.50, *SD* = 11.60) with no difference between the abbreviated and full version scores (*p* = .38).

### Data Acquisition and Analyses

A Tobii X2-60 Hz eye tracker (Tobii AB, Stockholm, Sweden) recorded pupil diameter. Pictures were viewed from 57 cm on a 39.6-cm laptop monitor in a dark and quiet room. Data were analyzed using MATLAB (MathWorks, Version 8.5, Massachusetts, USA). We removed any pupil diameter increase or decrease of 0.375 mm within one data reading and deleted the first data point that followed missing data. Data were smoothed using a low-pass filter for a span of five readings (83 ms). Pupil size was determined by calculating the mean diameter across both eyes. Pupil diameter for each trial in the period 200 ms before stimulus onset was subtracted from subsequent pupil size to establish baseline-corrected pupil diameter (Snowden et al., 2016).

Trials with fewer than 50% data points were omitted. Participants were excluded if they had less than 50% valid data across all trials, leading to the removal of three participants for the affective images (*n* = 79), five for the affective sounds (*n* = 77), and four for the dynamic facial expressions (*n* = 78). Split-half reliability estimates using the Spearman–Brown correction revealed good internal consistency for mean pupil diameter across tasks (affective images, *r* = .95; affective sounds, *r* = .84; dynamic facial expressions, *r* = .86).

### Statistical Analyses

To identify modulation of pupil diameter, we calculated the EMPR: the differences in pupil diameter between the affective stimulus and the neutral stimulus. This was calculated over 1,000-ms epochs throughout stimulus presentation. To investigate the effects of psychopathy on the EMPR, we performed separate analyses of variance (ANOVAs) for Factor 1 and Factor 2. Each ANOVA included a between-subjects factor of psychopathy score (as a continuous variable) and within-subject factors of stimuli valence (e.g., negative and positive) and time (over each 1,000-ms epoch). Where interactions were found, they were further examined by planned correlations between psychopathy and EMPR (separately for each valence). Where a significant relationship between a PCL-R factor and EMPR was identified, we ran supplemental analyses separating the PCL-R factors down into their facet components.

To explore the role of potential confounding variables, we examined the influence of psychotropic medication dosage, age, IQ, and substance misuse (see the online supplemental materials). The inclusion of these variables did not alter the general pattern of findings in relation to our main hypotheses (see Analyses 1 in the online supplemental materials). We also examined significant relationships between PCL-R scores and EMPR scores by repeating the analyses using only patients without a diagnosis of psychotic disorder (see Analyses 2 in the online supplemental materials). Two-tailed statistical analyses were conducted throughout. We reported 95% confidence intervals (CIs) for all correlational analyses and 90% CIs for ANOVAs (Steiger, 2004).

## Results

The sample had a mean PCL-R total score of 20 (*SD* = 8.0), with 33% of the sample having a score greater than 25 and 15% a score greater than 30. PCL-R scores as a function of diagnosis are given in the online supplemental materials (see Table 1).

### Affective Images

**Figure 1 fig1:**
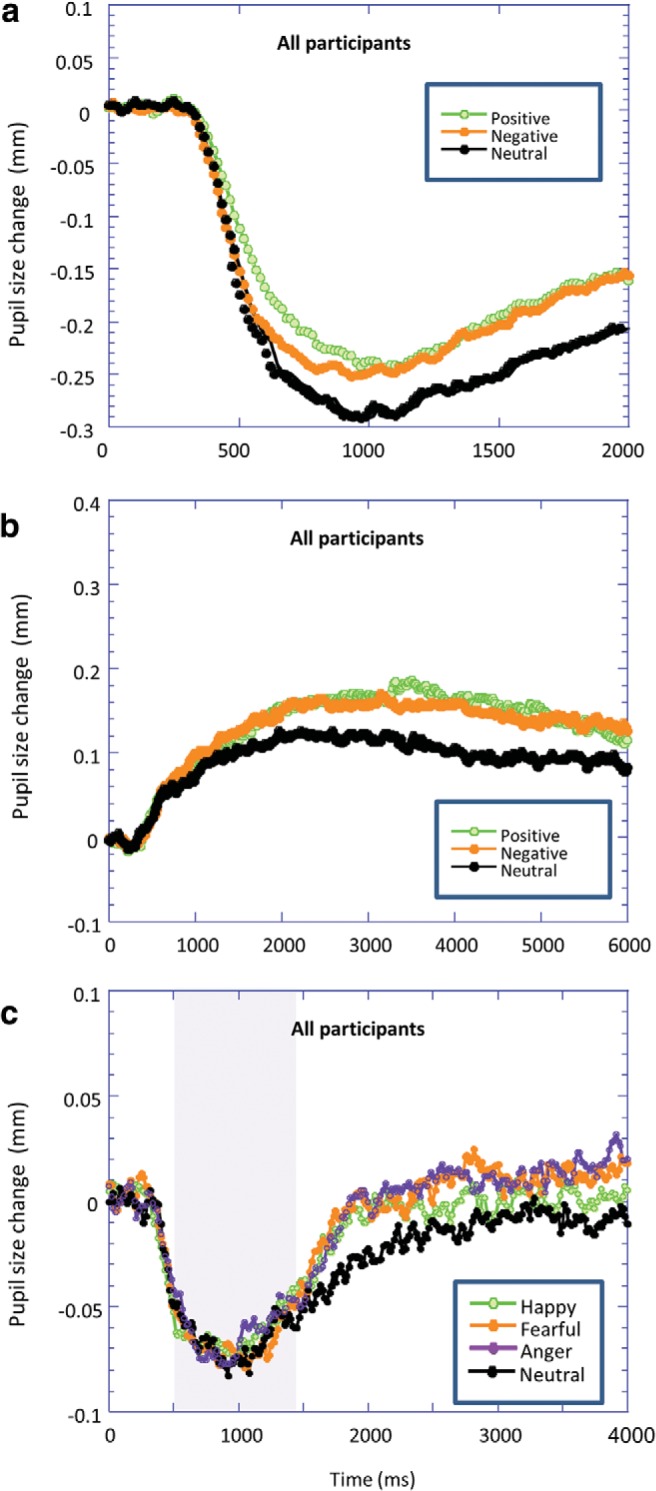
Baseline-corrected pupil diameter as a function of the time since stimulus onset in response to (a) images, (b) sound clips, and (c) dynamic facial expressions. Shaded area in part (c) represents period over which facial expression changes from neutral to expressive.

Figure 1a shows the change in pupil size poststimulus presentation in response to the images. We observed a pupillary constriction reflex from around 300 ms, before larger pupil diameter emerged from around 500 ms in response to the negative and positive images relative to neutral images. As our major aim was to investigate whether this affective dilation was altered by psychopathic traits, we first examined if there were any changes in the pupil response to neutral stimuli as a function of any psychopathy score (factors and facets). None of these correlations approached significance (*p*s > .15). We then quantified the EMPR by calculating the difference between the pupil diameter to affective and neutral images.[Fig-anchor fig1]

Two ANOVAs (for Factor 1 and Factor 2 separately) on the EMPRs were conducted with factors of psychopathy score (as a continuous variable), valence (negative and positive), and time epoch (0–1,000 ms and 1,000–2,000 ms). For Factor 1, there was a significant three-way interaction, *F*(1, 77) = 6.40, *p* = .01, η^2^ = .08, 90% CI [.01, .18]. This interaction was examined by looking at the correlation between Factor 1 and EMPR for each time window. Factor 1 was inversely related to EMPR for negative images, *r*(79) = −.23, 95% CI [−.43, −.01], *p* = .02, for the 1,000–2,000 ms time window. No other correlations were significant. The supplementary analysis on only those patients without a diagnosis of psychosis (see the online supplemental materials) also showed a significant relationship for Factor 1 and negative images at 1,000–2,000 ms, *r*(39) = −.55, 95% CI [−.74, −.29], *p* < .001.

We also conducted a supplemental analysis separating the PCL-R Factor 1 down into its facet components. The interpersonal facet (Facet 1) was negatively correlated with EMPR for the negative images at 1,000–2,000 ms, *r*(79) = −.23, 95% CI [−.43, −.01], *p* = .04, whereas Facet 2 (Affective) produced a similar effect size, but this was not statistically significant, *r*(79) = −.18, 95% CI [−.39, .04], *p* = .11.

**Figure 2 fig2:**
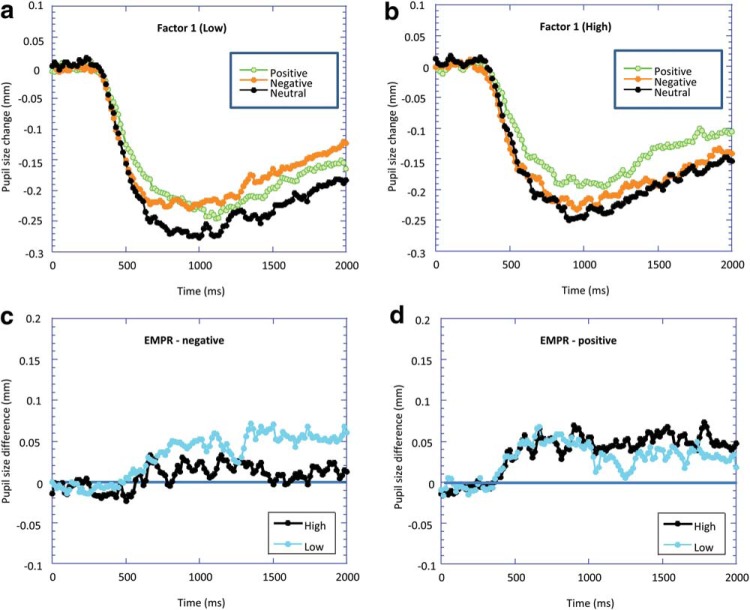
Baseline-corrected pupil diameter as a function of the time since stimulus onset in response to positive, negative, and neutral images for (a) high Factor 1 and (b) low Factor 1 groups. Emotional modulation of the pupil response (EMPR; the difference in pupil diameter to positive/negative images compared with neutral images) is plotted for both high and low Factor 1 groups for (c) negative images and (d) positive images.

To graphically illustrate these results, we split the participants according to Factor 1 score. The high Factor 1 group (*n* = 25) had a score of ≥10 (proportional to 25 for total PCL-R score; Cooke, Michie, Hart, & Clark, 2005), and the low Factor 1 group (*n* = 27) had a score of ≤4 (proportional to 10 for total PCL-R score). These groups approximately represented the top and bottom third of Factor 1 scorers. Figure 2a and b illustrates the pupil size change for each affective stimulus (negative, positive, and neutral) for the low Factor 1 and high Factor 1 scorers, respectively. We also calculated the EMPR for the negative and positive stimuli, and these are plotted in Figure 2c and d, respectively. For the low Factor 1 group, both the negative and positive images caused an increase in pupil size in comparison with the neutral images. For the high psychopathy group, the positive images caused pupil dilation in comparison with the neutral images, whereas the negative images did not. No main effects or interactions relating to Factor 2 were found.[Fig-anchor fig2]

### Affective Sounds

Figure 1b shows the change in pupil size poststimulus presentation in response to the sound clips, with larger pupil diameter observed in response to negative and positive sound clips. Psychopathy was not related to pupil response to the neutral sounds (*p*s > .15).

**Figure 3 fig3:**
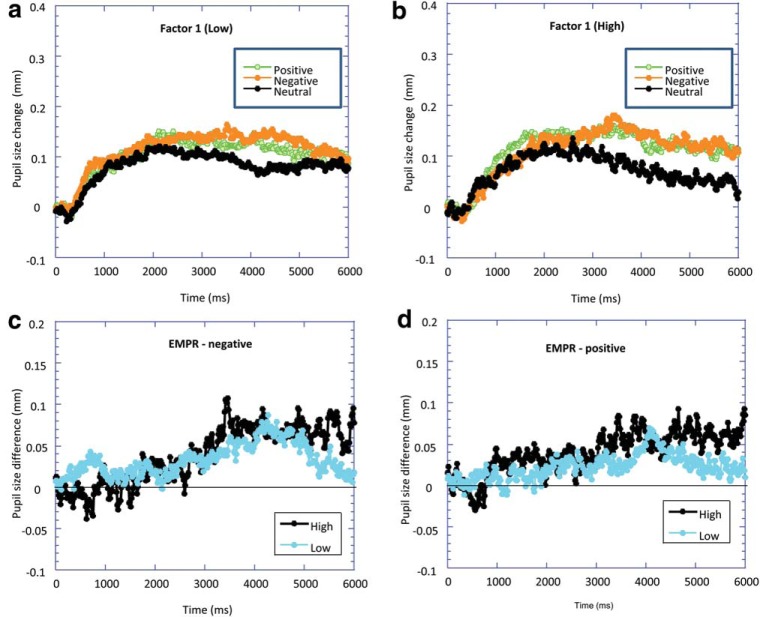
Baseline-corrected pupil diameter as a function of the time since stimulus onset in response to positive, negative, and neutral sounds for (a) high Factor 1 and (b) low Factor 1 groups. Emotional modulation of the pupil response (EMPR; the difference in pupil diameter to positive/negative sounds compared with neutral sounds) is plotted for both high and low Factor 1 groups for (c) negative sounds and (d) positive sounds.

We quantified the EMPR by calculating the difference between pupil diameter to the affective and neutral sounds. ANOVAs did not reveal any significant interactions between valence (negative and positive) and time for both Factor 1 and Factor 2, and so, no further analyses are reported. However, the data are illustrated in Figure 3a–d.[Fig-anchor fig3]

### Dynamic Facial Expressions

Figure 1c shows the change in pupil size poststimulus presentation in response to the dynamic faces. The initial presentation of the face produced the expected pupillary light reflex. The expression appeared on the face from around 500 ms and took up to 1,000 ms to complete (see shaded area), before greater pupil dilation to affective expressions emerged around 1,500 ms. To ease comparison with the other stimuli, we defined our time windows relative to the onset of the emotional expression (approximately 1,000 ms). There were no correlations (*p*s > .15) between any psychopathy measure and pupil response to the neutral stimuli.

For Factor 1, an ANOVA showed a significant interaction between valence (fear, happiness, and anger) and time on the EMPR, *F*(4.24, 301.31) = 4.40, *p* < .001, η^2^ = .06, 90% CI [.01, .09]. Factor 1 was negatively related to EMPR for the angry facial expressions over both the 0–1,000 ms, *r*(76) = −.24, 95% CI [−.44, −.02], *p* = .02, and the 1,000–2,000-ms time windows, *r*(76) = −.20, 95% CI [−.41, .03], *p* = .04. The supplementary analysis on only those patients without a diagnosis of psychosis (see the online supplemental materials) also showed a significant relationship for Factor 1 and angry expressions at 0–1,000 ms, *r*(39) = −.36, 95% CI [−.60, −.05], *p* = .03, and 1,000–2,000 ms, *r*(39) = −.33, 95% CI [−.58, −.02], *p* = .04. No correlations were significant for the fear expressions.

Finally, for the happy expressions, Factor 1 was positively correlated with EMPR in the 2,000–3,000-ms time window, *r*(76) = .34, 95% CI [.13, .53], *p* = .002. The supplementary analysis on only those patients without a diagnosis of psychosis (see the online supplemental materials) also showed a significant relationship for Factor 1 and happy expressions at 2,000–3,000, *r*(39) = .46, 95% CI [.17, .68], *p* = .004.

The facet analysis showed that the interpersonal facet (Facet 1) was negatively related to EMPR for angry faces over 0–1,000 ms, *r*(76) = −.24, 95% CI [−.44, −.01], *p* = .02, and 1,000–2,000 ms, *r*(76) = −.22, 95% CI [−.42, .01], *p* = .03. Facet 2 (Affective), however, only showed a trend-level relationship to EMPR at 0–1,000 ms, *r*(76) = −.19, 95% CI [−.40, .04], *p* = .05, and no relationship at 1,000–2,000 ms, *r*(76) = −.13, 95% CI [−.34, .10], *p* = .14. Both the interpersonal facet (Facet 1) and the affective facet (Facet 2) were positively associated with EMPR for happy faces over 2,000–3,000 ms, Facet 1: *r*(76) = .33, 95% CI [.12, .52], *p* = .003; Facet 2: *r*(76) = .29, 95% CI [.07, .48], *p* = .01.

**Figure 4 fig4:**
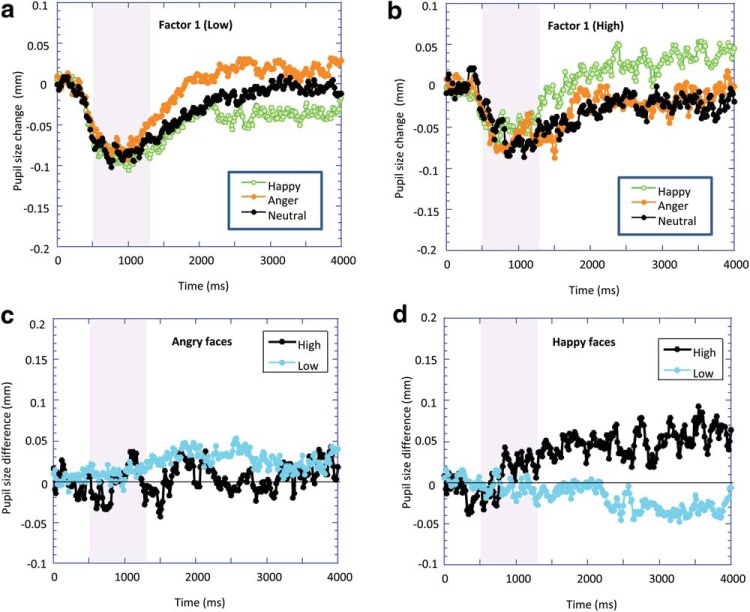
Baseline-corrected pupil diameter as a function of the time since stimulus onset in response to happy, anger, and neutral dynamic facial expressions for (a) high Factor 1 and (b) low Factor 1 groups (data from the fearful face are not plotted to simplify the figure, but followed the pattern for anger expressions closely). Emotional modulation of the pupil response (the difference in pupil diameter to happy/anger dynamic facial expressions compared with neutral dynamic facial expressions) is plotted for both high and low Factor 1 groups for (c) angry facial expressions and (d) happy facial expressions. Shaded areas represent period over which facial expression changes from neutral to expressive.

Figure 4a and b illustrates the results for low and high Factor 1 scores with the associated EMPRs in Figure 4c and d, respectively. For the low Factor 1 group, the angry stimuli produced the most pupil dilation (the fearful faces produced very similar results but have been omitted to improve the clarity of the figure), with the happy stimuli producing the least dilation. However, for the high Factor 1 group, there appeared to be little difference in pupil dilation in response to the angry faces compared with neutral, and it was the happy faces that produced the greatest dilation. The ANOVA for Factor 2 showed no significant effects.[Fig-anchor fig4]

### Time Course of Psychopathic Deficit

**Figure 5 fig5:**
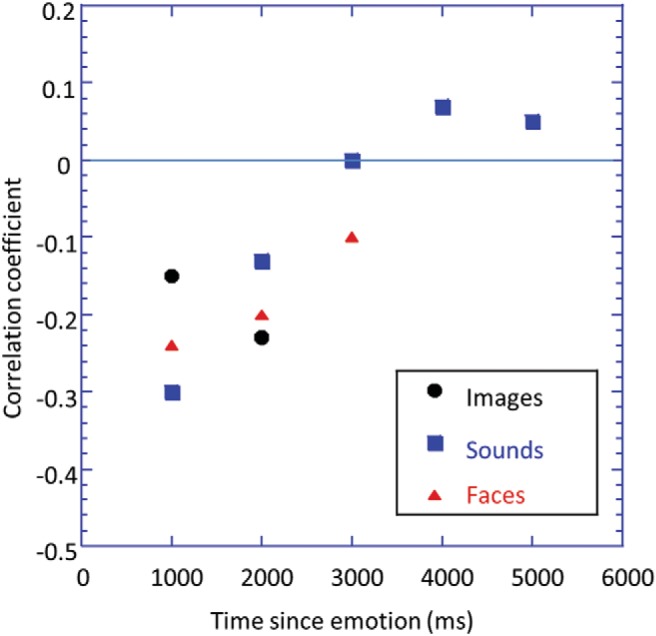
The correlation coefficient between Psychopathy Checklist—Revised Factor 1 score and the emotional modulation of the pupil response in response to negative stimuli across images, sound clips, and dynamic facial expressions (angry faces were used for the dynamic facial expressions) as a function of the time since emotional stimulus onset (the onset for the emotional dynamic faces was taken as 1,000 ms, which is approximately the midpoint of the transition from neutral to expressive).

We hypothesized that the effects of psychopathy might be greater during the early response to the affective stimulus. To test this idea, we plotted (see Figure 5) the correlation coefficient between Factor 1 score and the EMPR for the negative stimuli across the three experiments (angry expressions were used for the facial stimuli) as a function of the time since emotional stimulus onset. The correlation coefficients were consistently negative for time epochs of 2,000 ms and earlier, whereas the correlations were small for later periods.[Fig-anchor fig5]

## General Discussion

As expected, the pupil showed greater dilation when presented with emotional stimuli in comparison with neutral stimuli across all three types of stimuli. Our main hypothesis was that this emotional modulation would be reduced for individuals high in interpersonal–affective psychopathy. In line with this, individuals scoring high on Factor 1 (interpersonal–affective) of the PCL-R showed reduced pupil dilation to negatively valenced images. However, levels of psychopathy did not alter the response elicited by positive images. Factor 2 (lifestyle–antisocial) scores did not moderate pupil responses to affective images. A similar pattern of responses occurred in relation to the facial expressions, with clear evidence of reduced pupil dilation to angry faces for those with high Factor 1 scores. However, we did not show significant effects of psychopathy on the pupil response to auditory stimuli containing affective content.

### General Versus Negative-Specific Deficit

Our findings appear consistent with the Brook et al. (2013) review that asserted that across the diverging research literature on autonomic reactivity, “psychopathy appears to be associated with reduced autonomic responsiveness to negatively valenced/aversive stimulation” (p. 991). However, across varying paradigms (behavioral, psychophysiological, brain imaging, and self-report), Brook and colleagues also noted that psychopathy was at times associated with a generalized deficit across all emotions.

Perhaps the most germane research findings in respect to the present study are those that have examined other measures of sympathetic nervous system activity in response to affective stimuli. Some studies are supportive of the negative-specific deficit. For example, Benning et al. (2005) used affective images and found that individuals high in interpersonal–affective psychopathy traits showed normal SCRs to positive images, but smaller SCRs to negative images, in a community sample where psychopathy was defined via self-report. In contrast, Verona et al. (2004) measured SCRs to affective sounds (from the IADS) within an offender sample. They found that those high on Factor 1 showed a reduced response to all stimuli (including the neutral stimuli) and reduced differentiation between affective versus neutral stimuli, which was similar in magnitude for both negatively and positively valenced sound clips. Clearly, this result, a deficit for both negative and positive stimuli, contrasts with the present findings. The findings of Verona et al. (2004) are complicated by the overall lack of response in the high psychopathy group to the neutral stimuli, which makes comparison of low and high psychopathic groups hard to interpret. The present study does not seem to have this complication, as the autonomic responses to the neutral stimuli (across all three tasks) were not associated with any measure of psychopathy. For completeness, it should be noted that other studies have failed to find any effect of psychopathy on the emotional modulation of the SCR to affective stimuli (Pastor, Moltó, Vila, & Lang, 2003; Patrick et al., 1993), similar to the results found in the current study in response to auditory stimuli.

### Auditory Stimuli

The absence of a significant effect of psychopathy on pupil dilation to negative auditory stimuli is puzzling. Auditory stimuli from the IADS vary in intensity over time, and the nature of the affective content may take some time to become apparent. It may be that this “complexity” serves to reduce the psychopathic deficit by some mechanism. However, we note that for visual stimuli, the reduction in aversive-potentiated startle response due to psychopathy is greater for more complex stimuli (Sadeh & Verona, 2012). Alternatively, these auditory stimuli may be more “psychologically intense” than the visual stimuli and, therefore, able to overcome an emotion processing deficit. It might be possible to demonstrate a psychopathy-related deficit by using stimuli that are less intense or by distracting attention away from the auditory stimuli to reduce people’s ability to process these stimuli. Clearly, this is an area that requires further research.

### Factors and Facets

The interpersonal–affective dimension of psychopathy has been suggested to reflect a dispositional fearlessness caused by an insensitive defensive motivation system (Lilienfeld, Watts, Francis Smith, Berg, & Latzman, 2015) and appears hyporesponsive to negative stimuli. The association between reduced pupil responsivity, as indexed by emotional modulation to negative stimuli, and Factor 1, but not Factor 2, supports the idea of distinct factors underpinning psychopathy.

We also divided Factor 1 into the separate facets of interpersonal (Facet 1) and affective (Facet 2), according to the four-facet model of psychopathy (Hare, 2003). However, this analysis did not produce strong evidence that one of these facets was more powerful than the other in accounting for the hyporesponsivity to negative stimuli, although the results for Facet 1 (interpersonal) reached statistical significance, whereas those for Facet 2 (affective) did not. We note that Verona et al. (2004) showed a similar pattern where Facet 1 produced stronger modulation of SCRs than Facet 2.

### Time Course of Emotional Modulation

The time-sensitive response of the pupil allowed us to explore autonomic reactivity over the course of stimulus presentation. We observed reduced pupil responses to negative stimuli during the first 2,000 ms following the onset of the affective stimulus, with little evidence for deficits over later periods. This illustrates that there are greater effects of psychopathy early in the time window. Such a pattern of results suggests that the ability to process negative affect in psychopathy is not completely impaired, but rather takes more time or is delayed. Our results highlight that other researchers should consider the effects of time as a factor that might moderate possible deficits for those high in psychopathy.

### Pupil Responses to Happy Facial Expressions

We found evidence that the interpersonal–affective dimension of psychopathy (and the interpersonal and affective facets individually) was associated with increased pupil dilation to happy faces. Examination of Figure 4 shows that the happy faces produced a reduction in pupil size (in comparison with the neutral stimuli) for the low psychopathy participants but an increase in pupil size for the high psychopathy participants. This finding was unexpected. One possibility is that individuals who score highly on the interpersonal–affective dimension have a lack of interpersonal trust and could react suspiciously to the expression of someone smiling, thus causing arousal. Alternatively, we note that individuals who scored low in Factor 1 demonstrated an elevated pupil response to neutral compared with happy facial expressions. Previous research has shown that “neutral” faces are often perceived as somewhat hostile (Phillips et al., 1997). Hence, the greater pupil dilation observed to the neutral face than to the happy face for individuals scoring low in psychopathy may reflect a negative perception of this neutral face. Clearly, these ideas are speculative and the finding needs replication and further exploration.

### Limitations and Considerations

As is typical in forensic psychiatric settings, our sample had a variety of mental health diagnoses (see Table 1). Some of these diagnoses are associated with complex disturbances of emotional functioning that may serve to compromise our ability to isolate affective deficits specific to psychopathy (Taylor et al., 2012). For example, schizophrenia is associated with attenuated autonomic activity. We attempted to reduce such effects by only recruiting patients who were currently nonpsychotic. We also reran our statistical analyses using only participants without a history of psychotic disorder (see the online supplemental materials). The results of these analyses obtained similar (and even larger) effects of Factor 1 on pupil dilation for negatively valenced stimuli. Further, the same pattern of results occurred (see the online supplemental materials) when we statistically controlled for possible effects of psychotropic medication. We also note that previous drug and alcohol misuse cannot explain our current results (see the online supplemental materials).

A second limitation was that we used historically (up to 5 years old) gathered PCL-R information for around one third of the offenders, whereas the other PCL-R scores were assessed through an up-to-date file review. The use of historic measurement is not ideal. However, psychopathy scores on the PCL-R are fairly stable (Hare, 2003), and it seems unlikely that significant changes would occur within this period for a person in a secure setting. However, future studies should aim to calculate PCL-R scores using the same method for consistency.

## Conclusions

Using the rapid reaction of the pupil to stimuli with affective content, the current data support the hypothesis that psychopathy, specifically Factor 1 (interpersonal–affective), traits are associated with a deficit in processing negatively valenced affective information. Further, these effects of psychopathy were greatest shortly after the presentation of the affective stimuli, suggesting slowed affective processing rather than a global persistent insensitivity. No similar impairment was observed in response to positively valenced stimuli. Factor 2 (lifestyle–antisocial) scores were unrelated to the pupil’s response to emotional stimuli. The current pupillometry paradigm has obvious practical applications as a fast, time-sensitive, and nonintrusive measure to identify individuals who have problems in processing affective information.

## Supplementary Material

10.1037/per0000313.supp

## Figures and Tables

**Table 1 tbl1:** Detailed Demographic Description of the Participant Sample

Variable	*n*	%
Index offence		
Arson	6	7.3
Assault and grievous bodily harm^a^	26	31.7
Murder and manslaughter^b^	6	7.3
Sexual offenses	23	28.0
Theft	8	9.8
Other	13	15.7
Previous alcohol abuse	56	68.3
Previous substance abuse	53	64.6
Ethnicity		
White	77	93.9
Black	4	4.9
Asian	1	1.2
Mental health diagnoses^c^		
Schizophrenia and delusional disorders		
Schizophrenia	29	35.4
Persistent delusional disorder	4	4.9
Other	7	8.5
Mood disorders		
Bipolar affective disorder	2	2.4
Depressive disorder	4	4.9
Other	2	2.4
Neurotic, stress-related, and somatoform disorders		
Obsessive compulsive disorder	3	3.6
Posttraumatic stress disorder	4	4.9
Personality disorder		
Emotionally unstable personality disorder	19	23.2
Dissocial personality disorder	37	44.4
Other specific personality disorders	12	14.4
^a^ Including threats. ^b^ Including attempted murder. ^c^ All mental health diagnoses are reported, as many patients experienced co-occurring mental health disorders.		

## Footnotes

1 The responsible clinician has overall responsibility for care and treatment for service users being assessed and treated under the Mental Health Act.
