# Supplementary material for: Emotional Modulation of the Pupil Response in Psychopathy
Source: Personal Disord. 2018 Dec 3;10(4):365–75. doi: 10.1037/per0000313 (PMC6602524; doi:10.1037/per0000313)
Supplement: Supplementary file 1 [file PER-2018-1758_Supplementary.docx]

# Supplementary Analyses 1

We examined the influence of psychotropic medication dosage, age and IQ as potential confound variables in relation to PCL-R scores and our pupillary measures. We controlled for variables only that were associated to PCL-R scores *and* the relevant pupillary measure.

We converted anti-psychotic medication dosages into standard units of chlorpromazine according to equivalent dosages described by Andreasen, Pressler, Nopoulos, Miller, and Ho (2010). Anti-anxiety medication was quantified into standardised valium units according to equivalent dosages taken from the Benzodiazepine Equivalence Table (2007)^[[1]](#footnote-1)^. Moreover, anti-depressant medication was measured by converting dosages into standardised fluoxetine units according to equivalent dosages identified by Hayasaka et al. (2015)^[[2]](#footnote-2)^.

Chlorpromazine dosage was inversely associated with Factor 1, *r*(82) = -.36, *p* = .001, 95% CI [-.54, -.16], which extended to both the interpersonal facet (Facet 1), *r*(82) = -.32, *p* = .003, 95% CI [-.50 -.11], and the affective facet (Facet 2) respectively, *r*(82) = -.33, *p* = .003, 95% CI [-.51, -.12]. Chlorpromazine dosage was only related (inversely) to the difference between happy and neutral sound-clips from 1000 – 2000 ms, *r*(71) = -.28, *p* = .02, 95% CI [-.48, -.05], but Factor 1 continued to be unrelated to the difference in pupil diameter to happy - neutral sound-clips over 1000 – 2000 ms when chlorpromazine was controlled for, *r*(68) = -.08, *p* = .51, 95% CI [-.29, .18].

Valium dosage was positively related to Factor 2, *r*(82) = .27, *p* = .01, 95% CI [.06, .46], which extended to the antisocial facet (Facet 4), *r*(82) = .27, *p* = .01, 95% CI [-.06, .46], and the lifestyle facet (Facet 3), although this was only at a trend level, *r*(82) = .20, *p* = .07, 95% CI [-.02, .40]. Across the different stimuli, valium dosage was associated only to the difference in pupil diameter between angry and neutral facial expressions over each time-window, *r*s(76) > .34, *p* < .003. When controlling for valium dosage, Factor 2 continued to be unrelated to the difference in pupil diameter between angry and neutral facial expressions over each time-window (*p*s > .54). As an additional check of our main hypothesis, the findings for angry facial expressions in relation to Factor 1 (and the interpersonal facet [Facet 1]) were unaltered by controlling for valium dosage. Interestingly, when controlling for valium dosage, the affective facet (Facet 2) now showed a significant inverse relationship with angry-neutral facial expressions for 1000 – 2000 ms, *r*(72) = -.24, *p* = .04, 95% CI [-.45, -.01].

Fluoxetine dosage (*p*s > .15) and the centred interactions between medication types were unrelated to PCL-R factor or facet scores (*p*s > .13), and so were not considered further.

Participant IQ (see main text for further details) was negatively related to Factor 2, *r*(82) = -.26, *p* = .02, 95% CI [-.45, -.05], which extended to the lifestyle facet (Facet 3), *r*(82) = -.26, *p* = .02, 95% CI [-.45, -.05], as well as showing relationships to several pupil diameter measures in response to the dynamic facial expressions. Specifically, IQ was inversely associated to the difference in pupil diameter for angry – neutral facial expressions for each time-window from 0 – 3000 ms, *r*s(76) = -.27 < -.23, *p* < .05, and for happy – neutral facial expressions for both time-windows between 1000 – 3000 ms, *r*s(76) = -.23, *p*s < .05. However, when participant IQ was controlled for, Factor 2 remained unrelated to differences in pupil diameter between angry – neutral or happy – neutral facial expressions, *r*s(73) = -.004 < .12, *p*s > .34. As an additional check of our primary hypothesis, the findings were unaltered when IQ was controlled for. Participant IQ was unrelated to the remaining pupillary measures (*p* > .09),

Lastly, participant age was positively related to Factor 1, *r*(82) = .29, *p* = .01, 95% CI [.08, .48], but showed no significant association to the pupillary measures across experiments (*p*s > .08) and so was not considered further.

Interpersonal-affective psychopathy traits were associated with autonomic hypo-responsivity to negative stimuli largely independent of the influence of psychotropic medication across each task. However, it is useful to consider the method that was used to control for psychotropic medication. Specifically, psychotropic medication dosage was controlled for if the active metabolite was within the maximum half-life window. This quantified the dosage that the participant had taken over a longer period (usually within the last 24 hours), but this cannot inform regarding the exact amount of metabolite active during the research session. A more specific measure of psychiatric medication dosage would be useful. However, even if the specific amount of active metabolite could be quantified accurately it would be difficult to determine the specific influence of the medication, given that each individual is affected by psychotropic medication to differing degrees. Furthermore, while the interaction between anti-psychotic, anti-anxiety and anti-depressant medication was explored, it was not possible to isolate the interaction between specific medication types. Overall, extensive efforts were made to control for the effects of psychotropic medication to protect the validity of the findings, but inevitably the effects of psychotropic medication cannot be completely precluded as more complex interactions may exist.

# Supplementary Analyses 2

Schizophrenia has been previously associated with attenuated autonomic activity to emotion, particularly negative affect (Taylor et al., 2012), which may be problematic within the current forensic psychiatric sample as this could account for the hypo-responsivity observed to negative emotion for PCL-R Factor 1 if this factor were associated with a greater prevalence of schizophrenia.

Table 1 indicates PCL-R scores as a function of diagnosis, and it was apparent that individuals with a diagnosis of a psychotic disorder (i.e. schizophrenia, schizotypal or delusional disorder) showed relatively lower Factor 1 scores than individuals without schizophrenia. This was an initial indication that any potential association between the presence of a psychotic disorder and hypo-responsivity to negative affect was unlikely to be accounting for the present findings in relation to Factor 1.

We further sought to establish whether the presence of a psychotic disorder could be considered as an artefact in the present study, which may have accounted for Factor 1 predicting hypo-responsivity to negative stimuli. We defined a sub-group of 42 participants within our sample that were not diagnosed with a psychotic disorder and conducted supplementary analyses exploring the relationship between PCL-R scores and pupil responses to affective stimuli across images (*n* = 40), sound-clips (*n* = 39), and dynamic facial expressions (*n* = 38) respectively (see Tables 2 – 4 below). Again, we reported the difference in pupil diameter between each affective and neutral stimuli to identify pupil dilation specific to emotionally-laden stimuli.

Factor 1 continued to be negatively predictive of pupil responsivity to negative images (see Table 2) and to angry dynamic facial expressions over 1000 – 2000 ms (see Table 4) respectively for individuals without a psychotic disorder. It is evident that the effects of this pupil hypo-responsivity to negative stimuli were stronger within individuals without a psychotic disorder and extended across both earlier and later time-windows. Factor 1 was unrelated to pupil responsivity to negative sound-clips over the early time-window within this subset of patients (see Table 3) likewise to the overall sample. The supplementary analysis has provided evidence that the effects obtained in the main analyses could also be found in the absence of a psychotic disorder.

Lastly, another potential confound is that drug and alcohol misuse affects autonomic responsivity (Walsh, Allen, & Kosson, 2007) as psychopathy is associated with drug and alcohol misuse. However, problems with substance misuse are typically found in relation to Factor 2 (Walsh et al., 2007). This was also the case in the present sample (as evident in Table 5) likely indicating that the effects of Factor 1 on EMPR were not related to previous substance misuse. Table 5 shows PCL-R scores as a function of the presence of previous alcohol and substance misuse. Participant’s previous alcohol and substance was categorised from their patient records under the categories of ‘no evidence of use’, ‘experimental’, ‘recreational – occasional’, ‘recreational – heavy’ or ‘dependent’. Participants categorised as ‘recreational – heavy’ or ‘dependent’ were defined as individuals with previous substance misuse. Factor 1 scores were lower (though not significantly so) in those who have previously abused alcohol, *t*(80) = 0.68, *p* = .50, and substances, *t*(80) = 0.65, *p* = .52. In contrast, Factor 2 scores were higher for individuals who have previously abused alcohol (although, again, not significantly), *t*(80) = -1.53, *p* = .13, but this difference did surpass statistical significance for those who have abused substances, *t*(80) = -2.76, *p* = .01.

| Table 1  *Summary of mental health diagnoses in relation to PCL-R scores* | | | | | | | |  |  |  |  |
| --- | --- | --- | --- | --- | --- | --- | --- | --- | --- | --- | --- |
|  |  | | PCL-R Total | | | Factor 1 | | | Factor 2 | | |
| Diagnosis | *n* | Mean | | SD | Mean | | SD | | | Mean | SD |
| 1 | 23 | 12.23 | | 6.53 | 3.64 | | 2.57 | | | 7.74 | 4.77 |
| 2 | 4 | 16.75 | | 4.74 | 7.90 | | 2.74 | | | 7.33 | 3.40 |
| 3 | - | - | | - | - | | - | | | - | - |
| 4 | 32 | 23.60 | | 6.62 | 8.77 | | 4.39 | | | 12.81 | 3.09 |
| 1 and 2 | 1 | 16.00 | | - | 1.00 | | - | | | 12.00 | - |
| 1 and 4 | 13 | 21.35 | | 7.91 | 8.10 | | 4.08 | | | 11.77 | 4.90 |
| 2 and 4 | 2 | 27.40 | | 13.58 | 12.55 | | 4.88 | | | 11.85 | 7.28 |
| 3 and 4 | 4 | 17.90 | | 8.21 | 5.58 | | 6.14 | | | 10.00 | 2.01 |
| 1, 2 and 4 | 1 | 16.00 | | - | 4.00 | | - | | | 12.00 | - |
| 1, 3 and 4 | 1 | 30.00 | | - | 13.00 | | - | | | 14.00 | - |
| 1 = Schizophrenia, schizotypal and delusional disorders  2 = Mood disorders  3 = Neurotic, stress-related and somatoform disorders  4 = Personality disorder  One participant was yet to receive a formal mental health diagnosis | | | | | | | | | | | |

| Table 2  *Summary of zero-order correlations run between Psychopathy-Checklist Revised (PCL-R) factor scores and pupil diameter in response to negative and positive images (minus pupil diameter to neutral images) for a sub-group of participants (n = 40) not diagnosed with a psychotic disorder.* | | | | |
| --- | --- | --- | --- | --- |
|  |  | Factor 1 (Interpersonal-affective) |  | Factor 2 (Lifestyle-antisocial) |
| Stimuli | Time-window (ms) | *r* [95 % CI] |  | *r* [95 % CI] |
| Negative | 0 – 1000 | -.37* [-.61, -.07] |  | -.36* [-.60, -.06] |
|  | 1000 – 2000 | -.55* [-.74, -.29] |  | -.31 [-.57, .002] |
| Positive | 0 – 1000 | -.19 [-.47, .13] |  | -.32* [-.57, -.01] |
|  | 1000 - 2000 | -.19 [-.47, .13] |  | -.21 [-.49, .11] |

* *p* < .05

[95 % CI], confidence intervals are presented for correlations

| Table 3  *Summary of zero-order correlations run between Psychopathy-Checklist Revised (PCL-R) factor scores and pupil diameter in response to negative and positive sound-clips (minus pupil diameter to neutral sound-clips) for a sub-group of participants (n=39) not diagnosed with a psychotic disorder.* | | | | |
| --- | --- | --- | --- | --- |
|  |  | Factor 1 (Interpersonal-affective) |  | Factor 2 (Lifestyle-antisocial) |
| Stimuli | Time-window (ms) | *r* [95 % CI] |  | *r* [95 % CI] |
| Negative | 0 – 1000 | -.06 [-.37, .26] |  | -.001 [-.32, .32] |
|  | 1000 – 2000 | -.01 [-.32, .31] |  | -.01 [-.32, .31] |
|  | 2000 – 3000 | .16 [-.16, .45] |  | .03 [-.29, .34] |
|  | 3000 – 4000 | .24 [-.08, .52] |  | .23 [-.09, .51] |
|  | 4000 – 5000 | .17 [-.15, .46] |  | .19 [-.13, .48] |
|  | 5000 – 6000 | .23 [-.09, .51] |  | .14 [-.18, .44] |
| Positive | 0 – 1000 | -.14 [-.44, .18] |  | -.18 [-.47, .14] |
|  | 1000 – 2000 | -.07 [-.38, .25] |  | -.26 [-.53, .06] |
|  | 2000 – 3000 | .06 [-.26, .37] |  | -.19 [-.48, .13] |
|  | 3000 – 4000 | .13 [-.19, .43] |  | -.09 [-.39, .23] |
|  | 4000 – 5000 | .04 [-.28, .35] |  | -.12 [-.42, .20] |
|  | 5000 – 6000 | .15 [-.17, .44] |  | -.12 [-.42, .20] |

* *p* < .05

[95 % CI], confidence intervals are presented for correlations

| Table 4  *Summary of zero-order correlations run between Psychopathy-Checklist Revised (PCL-R) factor scores and pupil diameter in response to fearful, happy and angry facial expressions (minus pupil diameter to neutral facial expressions) for a sub-group (n = 38) of participants not diagnosed with a psychotic disorder.* | | | | | |
| --- | --- | --- | --- | --- | --- |
|  |  | Factor 1 (Interpersonal-affective) |  | Factor 2 (Lifestyle-antisocial) |  |
| Stimuli | Time-window (ms) | *r* [95 % CI] |  | *r* [95 % CI] | |
| Fearful | 0 – 1000 | -.20 [-.49, .13] |  | .29 [-.03, .56] | |
|  | 1000 – 2000 | -.27 [-.54, .05] |  | .27 [-.05, .54] | |
|  | 2000 – 3000 | -.25 [-.53, .18] |  | .31 [-.01, .57] | |
|  | 3000 – 4000 | -.02 [-.34, .30] |  | .42* [.12, .65] | |
| Angry | 0 – 1000 | -.37* [-.62, -.06] |  | .30 [-.02, .57] | |
|  | 1000 – 2000 | -.36* [-.61, -.05] |  | .23 [.10, .51] | |
|  | 2000 – 3000 | -.33* [-.59, -.01] |  | .27 [-.05, .54] | |
|  | 3000 – 4000 | -.19 [-.48, .14] |  | .40* [.09, .64] | |
| Happy | 0 – 1000 | -.20 [-.49, -.13] |  | -.10 [-.41, .23] | |
|  | 1000 – 2000 | .19 [-14, .48] |  | -.002 [-.32, .32] | |
|  | 2000 – 3000 | .20 [-.13, .49] |  | .03 [-.29, .35] | |
|  | 3000 – 4000 | .46* [.17, .68] |  | .15 [-.18, .45] | |

* *p* < .05

[95 % CI], confidence intervals are presented for correlations

| Table 5  *Summary of alcohol/substance abuse in relation to PCL-R scores* | | | | | | | |
| --- | --- | --- | --- | --- | --- | --- | --- |
|  |  | PCL-R Total | | PCL-R Factor 1 | | PCL-R Factor 2 | |
| Variable | *n* | Mean | SD | Mean | SD | Mean | SD |
| Alcohol abuse | | | | | | | |
| Yes | 56 | 19.67 | 8.39 | 6.77 | 4.32 | 11.21 | 4.64 |
| No | 26 | 18.47 | 8.34 | 7.49 | 4.83 | 9.60 | 3.96 |
| Substance abuse | | | | | | | |
| Yes | 53 | 19.94 | 8.65 | 7.60 | 4.26 | 11.67 | 4.36 |
| No | 29 | 18.10 | 7.76 | 8.53 | 4.42 | 8.93 | 4.20 |

# References

Andreasen, N. C., Pressler, M., Nopoulos, P., Miller, D., & Ho, B.-C. (2010). Antipsychotic dose equivalents and dose-years: a standardized method for comparing exposure to different drugs. *Biological Psychiatry, 67*(3), 255-262. doi.org/10.1016/j.biopsych.2009.08.040

Benzodiazepine equivalence table. (2007). *Retrieved from http://www.benzo.org.uk/bzequiv.htm*.

Hayasaka, Y., Purgato, M., Magni, L. R., Ogawa, Y., Takeshima, N., Cipriani, A., . . . Furukawa, T. A. (2015). Dose equivalents of antidepressants: Evidence-based recommendations from randomized controlled trials. *Journal of Affective Disorders, 180*, 179-184. doi: 10.1016/j.jad.2015.03.021

Taylor, S. F., Kang, J., Brege, I. S., Tso, I. F., Hosanagar, A., & Johnson, T. D. (2012). Meta-analysis of functional neuroimaging studies of emotion perception and experience in schizophrenia. *Biological Psychiatry, 71*(2), 136-145. doi: 10.1017/j.biopsych.2011.09.007

Walsh, Z., Allen, L. C., & Kosson, D. S. (2007). Beyond social deviance: Substance use disorders and the dimensions of psychopathy. *Journal of Personality Disorders, 21*(3), 273-288. doi:10.1521/pedi.2007.21.3.273

1. Two patients were taking buspirone, which we were not able to convert to standardised units due to differing mechanism of action compared to benzodiazepines. However, we left the participants within the sample as buspirone is not associated with side-effects of sedation or cognitive and psychomotor impairment unlike benzodiazepines in general. [↑](#footnote-ref-1)
2. Three of the patients were taking citalopram, but no study to our knowledge has explored equivalent dosage for this medication and so we used an equivalent dosage of 30 mg (in relation to 40 mg of fluoxetine), which was based on the median of the recommended target dosage range (Hayasaka et al., 2015). [↑](#footnote-ref-2)
